# Human colon function ex vivo: Dependence on oxygen and sensitivity to antibiotic

**DOI:** 10.1371/journal.pone.0217170

**Published:** 2019-05-16

**Authors:** Luke A. Schwerdtfeger, Nora Jean Nealon, Elizabeth P. Ryan, Stuart A. Tobet

**Affiliations:** 1 Department of Biomedical Sciences, Colorado State University, Fort Collins, Colorado, United States of America; 2 Department of Environmental & Radiological Health Sciences, Colorado State University, Fort Collins, Colorado, United States of America; 3 Program in Cell and Molecular Biology, Colorado State University, Fort Collins, Colorado, United States of America; 4 School of Biomedical Engineering, Colorado State University, Fort Collins, Colorado, United States of America; National Institute for Agronomic Research, FRANCE

## Abstract

**Background:**

Human intestines contain a heterogeneous collection of cells that include immune, neural and epithelial elements interacting in a highly complex physiology that is challenging to maintain ex vivo. There is an extreme oxygen gradient across the intestinal wall due in part to microbiota in the lumen and close to the gut wall, which complicates the design of tissue culture systems. The current study established the use of an organotypic slice model of human intestinal tissue derived from colonoscopy biopsies to study host-microbial interactions after antibiotic treatment, and the influence of oxygen concentration on gut wall function.

**Methods:**

Organotypic slices from human colon biopsies collected during routine colonoscopy provided three-dimensional environments that maintained cellular morphology ex vivo. Biopsy slices were used to study impacts of oxygen concentrations and antibiotic treatments on epithelial proliferation rates, and metabolites from tissue culture supernatants.

**Results:**

Immune function was validated via demonstration of a T lymphocyte response to *Salmonella enterica* serovar Typhimurium. Following 24 h of *Salmonella* exposure there was a significant increase in CD3^+^ T-lymphocytes in biopsy slices. Metabolite profiling of tissue culture supernatants validated the influence of antibiotic treatment under varied oxygen culture conditions on both host and microbiome-mediated metabolism. Epithelial health was influenced by oxygen and antibiotic. Increased epithelial proliferation was measured in lowered oxygen conditions (1% = 5.9 mmHg) compared to atmospheric conditions standard at 5000 feet above sea level in Colorado (~17% = 100 mmHg). Antibiotic treatment reduced epithelial proliferation only in 5.9 mmHg oxygen cultured slices.

**Conclusions:**

A human colon organotypic slice model was established for applications ranging from gut epithelial proliferation to enteric pathogen influence on mucosal immune functions ex vivo. The results further support the need to account for oxygen concentration in primary tissue cultures, and that antibiotic use impacts gut-microbe-immune interactions.

## Introduction

Gastrointestinal issues send over 70 million Americans a year to a physician, often due to complex inflammatory diseases such as ulcerative colitis and Crohn’s disease [1]. Physiological mechanisms underlying these diseases are poorly understood [2]. Teasing apart the etiology of intestinal pathologies in humans requires the use of in vitro model systems to mimic in vivo gut physiology. Methods for culturing human colon explants go back over 40 years [3], however there were issues of tissue degradation over time. Difficulties with maintaining intestinal tissues ex vivo are due in part to the unique physiology of the human colon, including the oxygen gradient seen across the intestinal wall.

The gastrointestinal tract is a complex multicellular tube with varied cellular composition based on not only region (e.g. ileum vs ascending colon) but also location within each gut region. The cellular heterogeneity of the intestine contributes to immunological functions including pathogen surveillance, antigen presentation, and secretion of pro- and/or anti- inflammatory cytokines. Lamina propria also contains dense enteric neural cell populations comprised of enteric glial cells and neuronal fibers [4] with the former having been implicated in issues ranging from barrier maintenance [5] to Crohn’s disease and ulcerative colitis [6,7].

Adding to the complexity of the colonic wall, the colonic mucosa sits in close proximity to the microbial communities of the gut. Composed of thousands of distinct species, these organisms, and in particular the metabolites they produce, play key roles in regulating intestinal physiology and homeostasis [8]. Microbial metabolism in the colon is responsible for production of a wide array of metabolites, offering a measure of the functional output of the microbiome [9]. Short chain fatty acids (e.g., acetate) are among the most common, and are thought to play key roles in inhibiting pro-inflammatory cytokine production [10]. How microbial derived metabolites regulate tissue physiology in protective and detrimental manners is an active area of investigation [11].

While the microbiome is normally commensal, pathogenic bacterial species such as *Salmonella enterica* can invade the intestinal mucosa and cause infection [12]. Once inside the gut wall, *Salmonella* can cause immune responses through a number of mechanisms, including Toll-like and NOD receptor signaling. Such signaling leads to activation and expansion of mucosal T lymphocyte populations [13,14]. In the present study, this *Salmonella* response was used to validate the competence of biopsy slices to elicit an immune response in vitro.

A steep oxygen gradient is present across the intestinal wall [15]. The apical mucosa closest to the lumen maintains in vivo oxygen concentrations of 0.1–1% oxygen (~1–6 mmHg). Climbing rapidly across the intestinal wall, the oxygen concentration is ~6% (~42 mmHg) in the vascularized submucosa. The colonic muscle wall is the most well oxygenated region, with 7–10% (42–71 mmHg) oxygen concentrations [16]. Alterations in this oxygen gradient can lead to impacts on the composition of the gut microbiome both in vivo [17] and in vitro [18]. Commensal bacteria can impact mouse intestinal contractions [19] and expression of genes associated with inflammation and antigen presentation [20]. In addition to oxygen culture condition, antibiotic exposure (e.g. penicillin-streptomycin) can substantially alter microbiome composition [21,22] and microbial derived metabolites such as phenyllactate [23].

Several methods that have been employed to maintain human colon tissue in vitro regularly use explants or immortalized cell culture monolayers (e.g. caco-2) at ambient oxygen (~120–145 mmHg) [24,25,26] or higher (675 mmHg) [5,27] concentrations, in the presence of antibiotics. One system recapitulated an oxygen gradient across a caco-2 monolayer culture in a gut-on-a-chip system using only a subset of bacterial strains instead of native human microbiome [28]. Influences of varied oxygen culture conditions and antibiotic exposures on microbially derived metabolites impacting gut immunity and general tissue physiology are unknown. The current study presents a human colon tissue model system with a heterogeneous mucosal cellular population at physiologic oxygen tensions, and without antibiotics. The model maintains epithelial structure, components of the immune system in the form of T-lymphocytes, and a metabolically active microbiome for up to 3 days ex vivo. The results demonstrate impacts of oxygen concentration and antibiotic exposure on biopsy slice epithelial structure, cell turnover rates, and metabolome composition.

## Methods

### Biopsy collection

**Fig 1:**
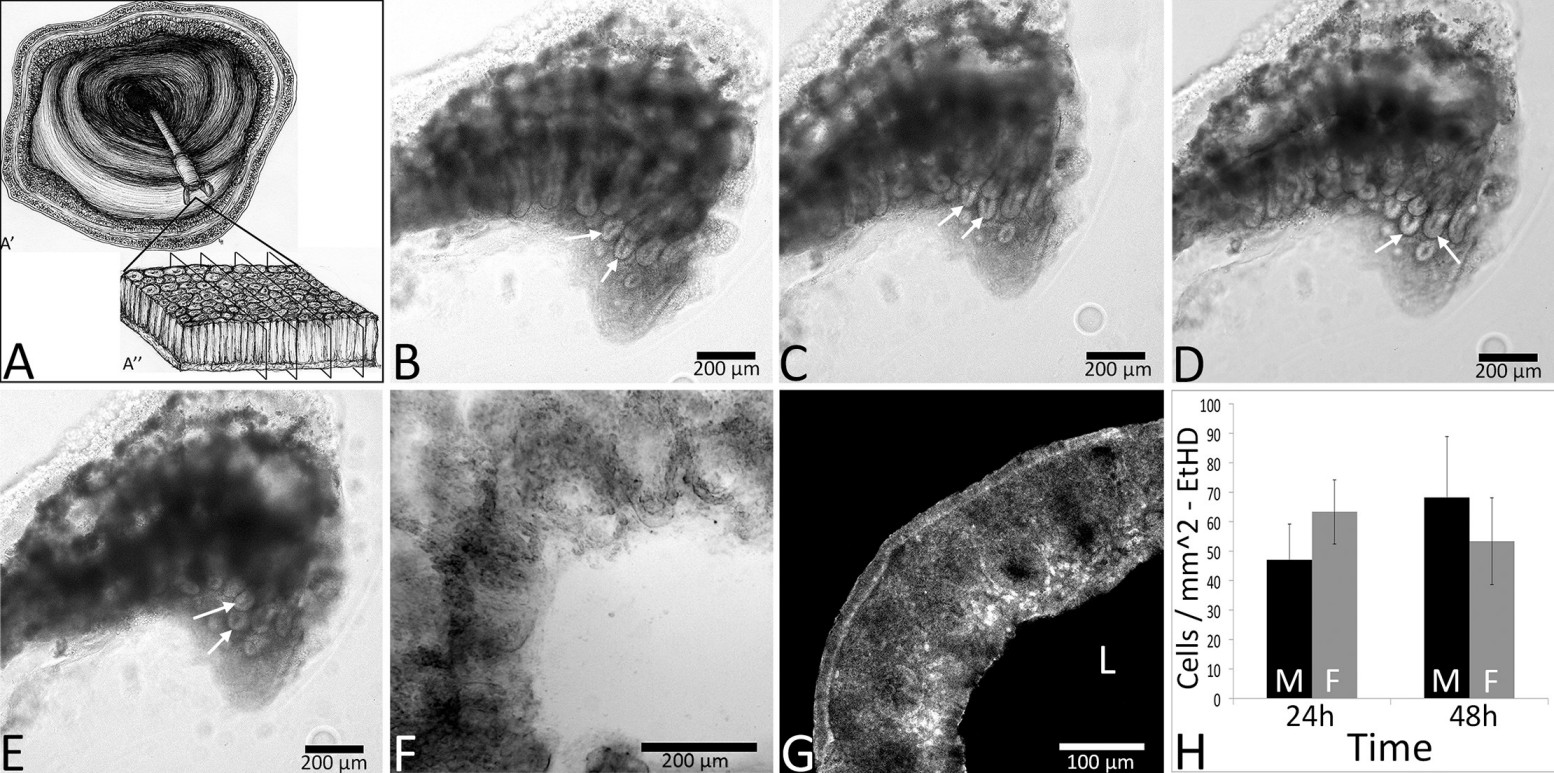
**Structural integrity of organotypic colon biopsy slices (schematic in A) was maintained for up to 3 days ex vivo.** Bright-field images of one organotypic biopsy slice at 0 (B), 24 (C), 48 (D) and 72h (E) ex vivo show intact colonic crypts and lamina propria. Crypt enterocytes were organized at the luminal surface (arrows). A representative slice is shown at 96h ex vivo with tissue degradation that rendered crypt patterning difficult to identify (F). A representative image shows minimal cell death (EtHD label; RFP; G) at 48h ex vivo. No differences were seen in EtHD label between 24 and 48h of culture regardless of sex (H). Data in panel H are +/- SEM, n = 3 male and 3 female participants per time point. Black bars in panel H represent males (M), and gray bars females (F). ‘L’ represents the luminal aspect.

Healthy adult participants were recruited prior to a routinely scheduled colonoscopy at Harmony Surgery Center or Poudre Valley Hospital (Fort Collins, CO). For each participant, colon biopsies of ~5mm diameter were collected from both the right (ascending) and left (descending) colon using standard biopsy forceps (Fig 1A’), and subsequent slicing (Fig 1A”). In total, biopsies were collected from 31 participants (21 female and 10 male), with between 10–30 slices being generated per biopsy. Mean age of participants was 56.1 +/- 2.8 years for females, and 56.2 +/- 2.3 years for males. Body mass index (BMI) was similar between sexes, with male mean BMI 28.8 +/- 1.3, and female 27.3 +/- 1.5. Biopsies were harvested from mucosal tissue, composed of colonic crypts and lamina propria, and did not penetrate the muscle wall. De-identified biopsies were immediately placed into 1X Krebs buffer (in mM: NaCl, 126; KCl, 2.5; CaCl_2_, 2.5; NaH_2_PO_4_, 1.2; MgCl_2_, 1.2) and maintained on ice for transport to Colorado State University. For all participants, colon tissue samples had less than 30 min transit time between collection and processing in the research lab. This project was approved by the University of Colorado Health Institutional Review Board under IRB #15–6051, and Colorado State University IRB registration number 00010144.

### Organotypic slice preparation

Slice preparation was similar to that previously described in [19]. Biopsies were placed in 4°C 1X Krebs buffer and dissected free of any connective structure. The entire tissue was submerged in 8% agarose (Agarose LM; Gold Biotechnology, St. Louis, MO) for a total of 7 min: 5 min on a room temperature shaker, and 2 min in 4°C to polymerize. Tissues were cut on a vibrating microtome (VT1000S; Leica Microsystems, Wetzlar, Germany) at a thickness of 250 μm. Slices from both right and left colon biopsies were pooled. Slices were collected in 4°C 1X Krebs buffer, and immediately transferred into a 60 mm plastic-bottom dish (Corning, Corning, NY) containing 5 ml of Hibernate media (Life Technologies, Grand Island, NY) and either zero (nPS) or 1.3% penicillin-streptomycin (PS; HyClone Laboratories, Logan, UT). Slices spent at least 15 min in Hibernate media (Gibco, Grand Island, NY) at 4°C before being transferred to 5 ml of Adult Neurobasal Media (ANB; Life Technologies) containing 5% B-27 supplement (B-27; Life Technologies). For slices treated with antibiotic, PS was added to the media (final concentration of 1.3%). Next, slices were transferred to a 37°C incubator for 35 min. Samples were then plated on 35 mm diameter plastic bottom dishes (Corning). Slices were allowed to adhere to the dish for 10 min at 37°C before being overlaid with a bovine collagen solution [vol/vol: 10.4% 10X MEM (Minimal Essential Medium, Sigma-Aldrich, St. Louis, MO), +/- 1.9% PS, 4.2% sodium bicarbonate, and 83.5% collagen (PureCol; Inamed, Fremond, CA)]. The collagen solution was allowed to polymerize in 37°C for 20 min before a final 1 ml addition of ANB with B-27 and +/- PS that was pre-incubated in either a standard 37°C, 5% CO_2_, ambient oxygen incubator (100 mmHg), or a three gas incubator set for 37°C, 5% CO_2_, and 5.9 mmHg oxygen as modulated by nitrogen injection (Panasonic MCO-5M-PA; Panasonic Healthcare, Tokyo, Japan). Finally, slices were left at 37°C in either ambient or 5.9 mmHg oxygen until visualization or experiments as described below. All slices generated from a given biopsy were used for experimentation, however when a rare slice (< 5% of slices) had large amounts of cell debris in the luminal aspect and un-patterned crypts with an undefined tissue edge, it was discarded. Experiments were performed on slices after at least 24 h of culture, and no longer than 72 h of culture. This ensured that slices were able to recover fully after cutting procedures prior to experimentation (24 h), and also that slices were not undergoing tissue degradation prior to experiments concluding (72 h).

### Live slice imaging

Slices were imaged on one of two microscopes: a Nikon TE2000-U inverted microscope (4X, 10X Plan-Fluor and 20X Plan-Apo objectives) with a UniBlitz shutter system (Vincent Associates, Rochester, NY) and an Orca-flash 4.0 LT camera (Hamamatsu, Hamamatsu City, Shizuoka Prefecture, Japan), or a Zeiss LSM 800 confocal microscope with an Axiocam 503 mono camera (Carl Zeiss, Inc., Thornton, NY).

### Salmonella inoculation

Organotypic colon biopsy slices were challenged with Green Fluorescent Protein (GFP) tagged *Salmonella enterica* serovar Typhimurium (*S*. ser. Typhimurium-GFP; strain 14028s) which was generously donated by Dr. Andres Vazquez-Torres (University of Colorado) and prepared as previously described [29]. Briefly, *S*. ser. Typhimurium-GFP was brought up in Luria Bertani Broth (Mo Bio Laboratory, Carlsbad, CA) to 1x10^8^ colony forming units (CFU’s) per 1 ml. 1 μl of *S*. ser. Typhimurium-GFP or vehicle (sterile Luria Bertani broth) was added adjacent to the luminal aspect of nPS biopsy slices under 100 mmHg oxygen conditions, due to the strict aerobic nature of this strain. Invasion into the colonic epithelium was visualized using a Nikon inverted microscope setup for time-lapse video microscopy with a 488 nm excitation wavelength. Time-lapse video microscopy was performed at 0 h post inoculation and again at 4 h, for 10 min each with 30 s between exposures. After 4 h, any non-tissue adherent *S*. ser. Typhimurium-GFP was washed away with 3x media (ANB + B27) washes, and allowed to incubate for an additional 20 h prior to fixation followed by immunohistochemistry for CD3^+^ T-lymphocytes.

### Whole-mount immunohistochemistry (IHC)

Following live culture, slices were immersion fixed in 4% formaldehyde (Polysciences, Inc. Warrington, PA) for 15 minutes and washed three times in 0.05 M PBS, pH 7.5. Immunohistochemical studies were performed similar to those previously described [19]. Post-fixing and PBS washes, slices were incubated at 4°C for 2 h in 1% sodium borohydride in PBS. Slices were then washed two times in PBS for 5 minutes and subsequently incubated in a blocking solution composed of PBS, 5% normal goat serum (NGS; Lampire Biological, Pipersville, PA), 3% hydrogen peroxide and 0.3% Triton-X (Tx) for 1 h before a change into fresh solution for subsequent 1 h. Slices were then placed into affinity purified polyclonal anti-CD3 (cell surface marker for T lymphocytes; Dako Denmark, Glostrup, Denmark), affinity purified polyclonal anti-ZO-1 (tight junction protein; Invitrogen, Eugene, OR), polyclonal anti-peripherin (peripheral neuronal marker; EMD Millipore, Billerica, MA), or monoclonal anti-S100β (glial cell marker; Abcam, Cambridge, MA), composed of PBS with 5% NGS and 0.3% Tx for 4 days. Anti-CD3 was used at a concentration of 2 μg/ml, anti-ZO-1 at 2 μg/ml, anti-peripherin diluted 1:300, and anti-S100β at 2.4 μg/ml. Samples without primary antibody were used as control. After 4 days of incubation with primary antibody, slices were washed at 4°C with PBS containing 1% NGS and 0.02% Tx for 2 h with 4 changes. Next, biotinylated secondary anti-serum (anti-rabbit, 1:2500 for ZO-1, peripherin, S100β, and anti-mouse, 1:1000 for CD3; Jackson ImmunoResearch, West Grove, PA), specific to the species of the primary antibody, was made up in PBS with 1% NGS and 0.32% Tx and added overnight. Lastly, tissue was washed 4 times at room temperature in PBS with 0.02% Tx before being placed into solution containing the conjugated fluorophore (Cy-3 Streptavidin) for 3 h before being washed in PBS for 2 h and subsequently mounted on slides and cover slipped with an aqueous mounting medium (Aqua-Poly/Mount, Polysciences, Warrington, Pa). Following the IHC protocol, tissue was imaged on either a Nikon TE2000 inverted microscope, or a Zeiss LSM 800 confocal microscope (Carl Zeiss, Inc., Thornton, NY). A researcher blinded to treatment group counted cells using the ImageJ analysis software ‘analyze particles’ toolset.

### Metabolite profile analysis of supernatants

#### Data processing and metabolite identification

Metabolomics was performed by Metabolon Inc (Durham, NC, USA). Tissue culture supernatants from ex vivo tissue slices (4 females, 4 males) were collected in triplicate and stored at -80° C until processing. Supernatants were garnered from slices that were not treated with *S*. ser. Typhimurium-GFP. Processing of supernatants was performed with an 80% methanol extraction and metabolite detection via ultra-high-performance liquid chromatography-tandem mass spectrometry. Raw data was processed by Metabolon Inc as described previously [30], where it was extracted from the mass spectrometer, quality-controlled, and raw peaks and retention times were aligned across individual samples. With the aligned data, compound identifies were confirmed within an internal Metabolon library containing over 3,300 purified standards by comparing retention times and mass/charge ratios to those of purified standards. Retention time indices had to match within a narrow window, their mass to charge ratios had to include a mass within +/- 10 parts per million of a library entry, and their mass spectral profiles had to contain forward and reverse match scores that fell between the experimental data and spectral profiles from the Metabolon database. For each metabolite, total ion current area under the curve quantitation was used to generate metabolite raw abundances. These raw abundances were log2-transformed and median-scaled by dividing the metabolite’s raw abundance by the median raw abundance of that metabolite across the entire data set. For any sample missing a metabolite, the minimum median scaled abundance was input. For each metabolite, fold differences were determined between pairs of treatments by dividing the average median scaled abundance of a metabolite across one treatment group by another. Pairs of treatments used to calculate fold differences included: 100 mmHg O2, antibiotic; 100 mmHg O2, no antibiotic; 5.9 mmHg O2, antibiotic; 5.9 mmHg O2, no antibiotic.

#### Pathway enrichment score calculations

Following annotation, metabolites were grouped into pathways based on their biological/biochemical functions. Pathway enrichment scores (PES) were calculated to assess the overall contribution of a pathway to explaining treatment differences between 100 mmHg and 5.9 mmHg oxygen in both the presence and absence of antibiotics. PES were calculated using the following equation:

$$\frac{\left(\frac{k}{m}\right)}{\left(\frac{n}{N}\right)}$$

Where “k” represents the number of statistically-significant metabolites in the pathway, “m” is the total number of metabolites in the pathway, “n” is the number of statistically-significant metabolites across all pathways, and “N” is the total number of detected metabolites. PES greater than one are considered major contributors to treatment differences. GraphPad Prism Version 7.0 (San Diego, CA, USA) was used to visualize all PES.

### Cell proliferation analysis

Incorporation of the thymidine analog 5-ethynyl-2’-deoxyuridine (EdU; Invitrogen) was used to label cells undergoing DNA synthesis. After slices were plated, they were incubated at ambient oxygen, 5% CO_2_ or in an incubator with 5.9 mmHg oxygen, 5% CO_2_ for 24 h before the addition of EdU at a final molarity of 4 μM. Half of slices were subjected to PS treatment for the entirety of their time ex vivo and experimental process while the other half did not receive antibiotic. All slices were fixed at 48 h ex vivo in 4% formaldehyde prior to processing for visualization. Slices were washed 3 times in phosphate-buffered saline (0.05M PBS), followed by 30 min in glycine (Fisher Scientific, Pittsburgh, PA) at 4°C, and again washed with PBS for 10 min with 1 change. Slices were then blocked in 3% bovine serum albumin buffer (BSA; Lampire Biological, Pipersville, PA) and 0.5% Tx for 2 h. Post blocking, tissue was washed twice with 3% BSA before addition of Click-IT cocktail (1X click-It Reaction Buffer, CuSO_4_, Alexa-Fluor azide, 1X reaction buffer additive; Invitrogen) for 2 h at room temperature. Finally, slices were washed 3 times in 3% BSA with 0.02% Tx for 30 min each, and left in a final wash of 3% BSA prior to imaging. Analysis of EdU incorporation was performed using ImageJ Image Processing and Analysis software (NIH; Version 1.49) to determine the percentage of area labeled, with the whole biopsy slice as the region of interest. EdU incorporation was determined to be cellularly localized based on the cell size and anatomical localization in the colonic crypts. Using ImageJ, regions of interest (ROIs) were chosen based on crypt anatomy, with ‘Crypt’ ROIs encompassing one crypt region in that plane, and ‘nCrypt’ ROIs being similarly sized regions not containing any portion of a crypt. Optical density was used to set image thresholds, and small objects were eroded to remove spurious signals, and measured using the ‘analyze particles’ tool. A researcher blinded to treatment condition counted cells contained within ROIs.

### Cell death analysis

The incidence of cell death was measured using Ethidium Homodimer (EtHD; Biotium, Hayward, CA), a membrane impermeable, red fluorescent (RFP), DNA marker. EtHD was added to slice dishes containing three slices each, ex vivo, for 45 minutes, at a volume of 1 μl of 2.5 mM stock EtHD per 1 ml of media (ANB + B-27 ± PS). This resulted in a final concentration of 2.5 μM of EtHD, before the unbound material was washed out. EtHD labeling was analyzed using ImageJ (NIH; Version 1.49). One image per slice, totaling three images per biopsy were taken prior to thresholding according to optical density. Subsequently, objects smaller than 10 μm^2^ were deemed too small to be cells and were eroded to remove spurious signals. Finally, images were measured using the ‘analyze particles’ tool to garner cells counts. All data were collected by a researcher blinded to treatment condition.

### Statistics

All slices were generated from two biopsies from the same colon as biological replicates, and slices were used in triplicate to generate technical replicates, prior to statistical analyses being performed with the number of patients = n for analysis. T- lymphocyte data were analyzed by repeated measures ANOVA that accounted for pathogen exposure and sex of the tissue donor. For all metabolite analyses, statistics were performed by Metabolon Inc using Array Studio (Omicsoft, Cary, NC, USA). Briefly, a 2-way ANOVA was applied across all metabolite abundances over the treatment groups described above with a Welch’s post-hoc test. To account for multiple comparisons, a false discovery rate (q-value) was calculated for each metabolite across each contrast. Given the small number of samples and the large number of metabolites, there was insufficient power to generate statistically reliable sex differences. The metabolite data presented is combined for males and females. Cell proliferation data were analyzed by repeated measures ANOVA that accounted for oxygen condition, sex of tissue donor and antibiotic treatment, with antibiotic treatment as a repeated measure. Finally, cell death data were analyzed by 2-way ANOVA that accounted for sex of tissue donor and time point as a repeated measure. A p-value < 0.05 was considered as statistically significant in all analyses.

## Results

### Tissue integrity was maintained for 72 h (3 days)

**Fig 2:**
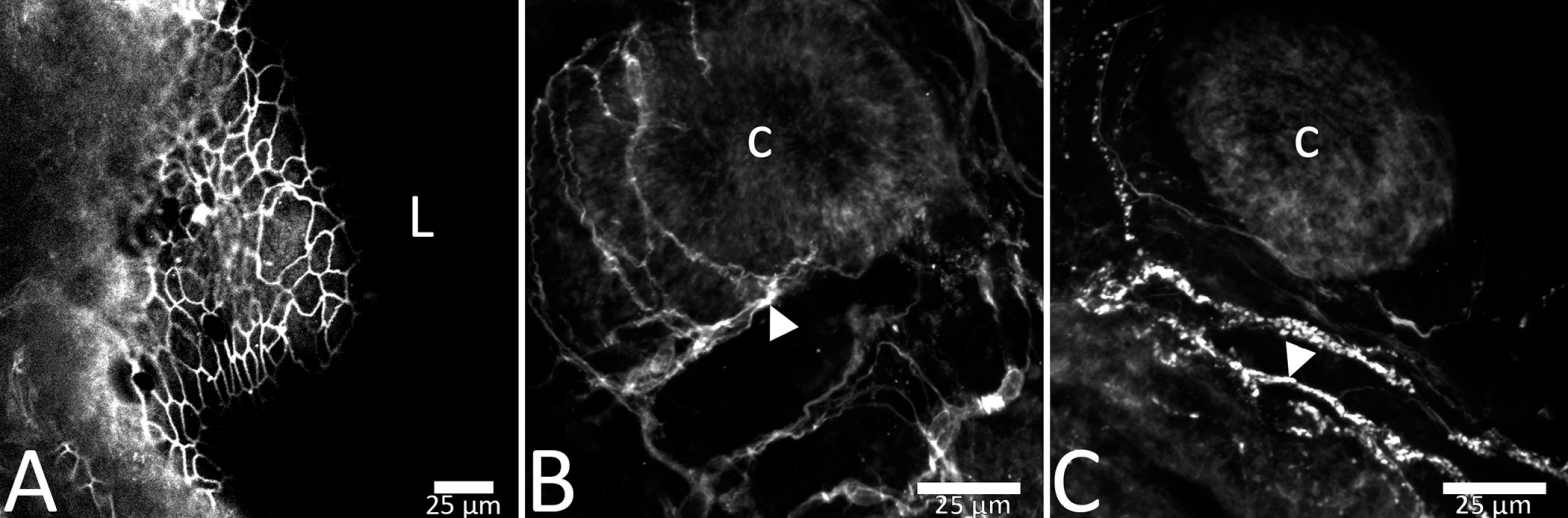
**Barrier integrity was intact at 24 hours ex vivo, as assessed by the presence of tight junction protein ZO-1 at the apical mucosal surface (A).** Neural components of lamina propria were present at 24h ex vivo. Representative images show S100β-immunoreactivity at 24h ex vivo indicating enteric glial cells (B) and peripherin immunoreactivity indicating neuronal fibers ex vivo (C). Scale bars in panels A–C are 25 μm. ‘L’ represents the luminal aspect and ‘c’ represents a colonic crypt. Arrowheads in panels B and C represent fibers with stereotypic immunoreactivity.

There was strong evidence of morphological preservation from freshly collected biopsy tissue in the form of patterned rows of crypts with minimal cell debris and a defined tissue edge. Cell turnover was also observed in organotypic slices for up to 3 days *ex vivo*. In 250 μm slices at 24, 48, and 72 h, colonic crypts were intact and patterned in rows and columns, and showed organized tissue at the crypt surface (Fig 1B–1E). At 96 h ex vivo, there was degradation of observable patterned rows of crypts in many slices (Fig 1F) indicating a decline in tissue viability. The apical epithelia were organized with tight junction protein ZO-1 present between epithelial cells at 24h (Fig 2A). Immunohistochemistry for enteric glial marker S100β and the neuronal intermediate filament protein peripherin show presence of neural components of the colonic mucosa at 24h ex vivo (Fig 2B and 2C). Intestinal function was further confirmed by measures of epithelial cell turnover using indicators of death (EtHD) and proliferation (EdU incorporation). Cells in slices died at a mean rate of 60 +/- 11.9 cells/mm^2^ across 24 h and 48 h ex vivo (less than 5% of tissue area; Fig 1H) consistent with the rate of cell turnover seen in the human colon in vivo [31].

### T–Lymphocytes increase in response to *S*. Typhimurium infection

**Fig 3:**
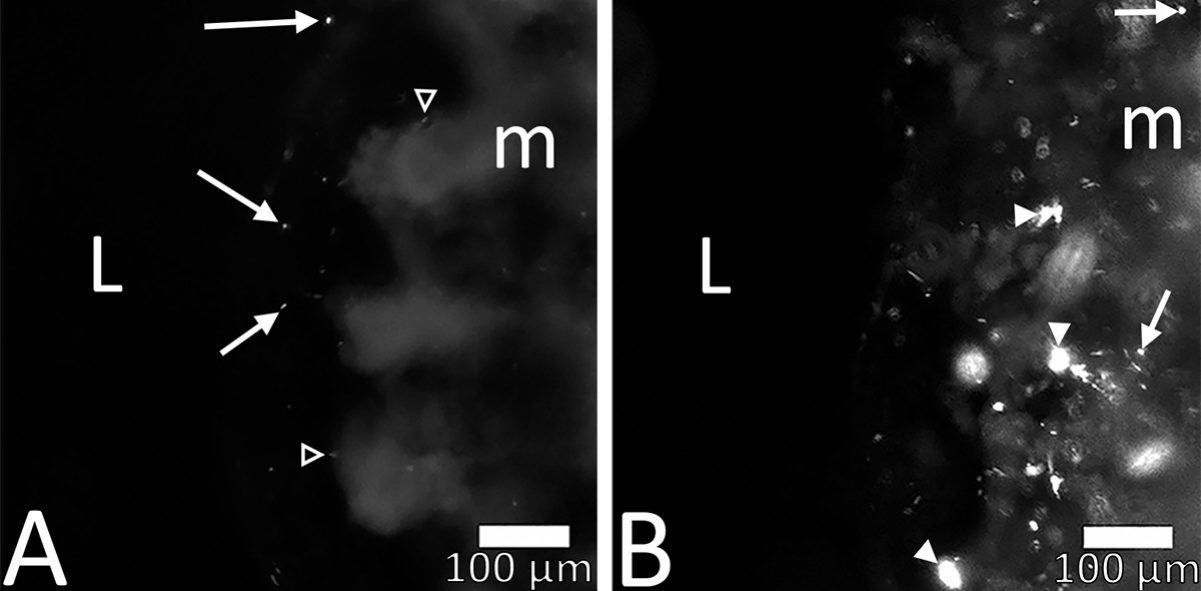
*S*. *ser*. Typhimurium-*GFP* infiltrated colon mucosa within 24 h of culture. At 4 h ex vivo (A) *S*. *ser*. Typhimurium-*GFP* were seen approaching the luminal aspect of the colonic mucosa (arrows) with minimal mucosal binding observed (open arrow heads in A). *S*. *ser*. Typhimurium-GFP appeared in colonic mucosa at 24 h post inoculation (B), and were often clustered together (arrowheads in B). Scale bar in both panels is 100 μm.

**Fig 4:**
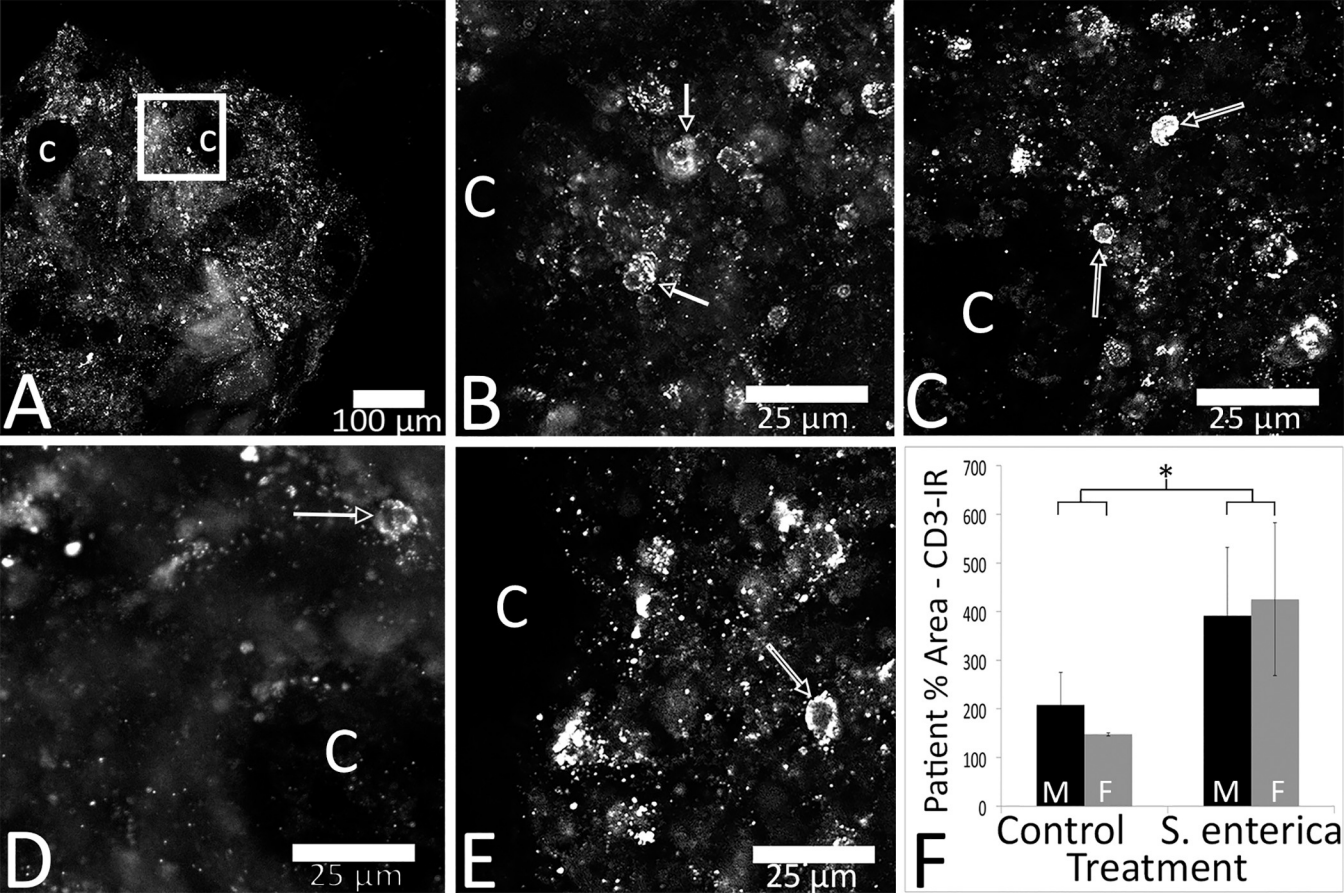
Challenge with *S*. *ser*. Typhimurium-*GFP* lead to an up-regulation of CD3^+^ T-lymphocytes after 24 h, regardless of sex. Images of CD3-IR (RFP in A-E) were captured from a region of lamina propria close to a colonic crypt. An example region of interest where higher magnification images were acquired is represented with a white square in (A) that applies conceptually to B-E. Representative images of CD3-IR in slices show less immunoreactivity in control (B, male; D, female) versus *S*. *ser*. Typhimurium-*GFP* treated slices (C, male; E female). There was a significant impact of treatment on CD3-IR, regardless of sex (F). Black bars represent males (M) and grey bars females (F). L = lumen, m = mucosa, C = colonic crypt. Arrows point to individual bacterium. Open arrows point towards cells with stereotypic CD3-IR. * Signifies p < 0.05. Data in panel I are +/- SEM, n = 4 male and 3 female participants.

T-lymphocyte counts increased in biopsy slices after a 24 h challenge with *S*. *ser*. Typhimurium-GFP. After 4 h, fluorescence was visible in the apical most regions of colonic mucosa (Fig 3A), primarily as scattered individual fluorescent particles. At 24 h post inoculation, there were notably more *Salmonella* seen throughout the colonic mucosa, particularly in clusters (Fig 3B, arrow heads), but with some bacteria still independently bound to the mucosa (arrows). After slice fixation and subsequent IHC for CD3, biopsy slices showed CD3^+^ cells localized closely with tissue adherent *S*. ser. Typhimurium-GFP (Fig 3C). Fig 4A presents a visualization of the ROIs used for generating CD3-IR data. After biopsy slices were challenged with *S*. *ser*. Typhimurium-GFP, immunoreactive (IR) T-lymphocytes were increased in slices from both males (Fig 4B, control; 4C, treatment) and females (Fig 4D, control; 4E treatment) 24h later (48h ex vivo; Fig 4F). There was a significant increase in T-lymphocytes following pathogen challenge, independent of sex [F(1,5) = 7.480; p < 0.05)].

### Metabolites and metabolic pathways were impacted by oxygen concentration and antibiotic presence

There were 258 metabolites identified in the tissue culture supernatants maintained for 48 h ex vivo from 31 colon slices obtained from 8 participants. Table 1 provides an overview of the number of metabolites in each of the chemical classes detected, including: amino acid (119), peptide (3), carbohydrate (13), energy/tri-carboxylic acid cycle (9), lipid (46), nucleotide (24), cofactors and vitamins (20), and xenobiotics (24). Statistically significant [p < 0.05] changes were identified based on the concentration of oxygen in the tissue culture incubator (5.9 mmHg oxygen versus 100 mmHg oxygen) as well as due to the presence or absence of antibiotic in the tissue culture media. Across oxygen concentrations, independent of antibiotic status, there were 41 metabolites that were significantly different (Table 1). When comparing antibiotic treatments, independent of oxygen concentration, 198 metabolites differed (Table 1). In addition, there was an interaction between antibiotic and oxygen status for 28 metabolites (Table 1). A complete list of identified metabolites, including those that were statistically different when accounting for oxygen concentration, antibiotic presence, and for oxygen*antibiotic are provided in the supporting information (S1 Table).

**Table 1:** Number of identified metabolites from tissue culture supernatants across oxygen and antibiotic treatments, organized by chemical class.

Chemical Class	100 mmHG Oxygen, Antibiotic	100 mmHG Oxygen, No Antibiotic	5.9 mmHg Oxygen, Antibiotic	5.9 mmHg Oxygen, No Antibiotic
Amino acids	116	118	110	118
Peptides	3	3	3	2
Carbohydrates	12	13	12	12
Energy/Tricarboxylic Acid Cycle	8	9	9	8
Lipids	46	46	39	45
Nucleotides	22	24	21	24
Cofactors/Vitamins	20	20	19	20
Xenobiotics	22	20	18	22
**Total number of identified metabolites**	249	253	231	251

**Fig 5:**
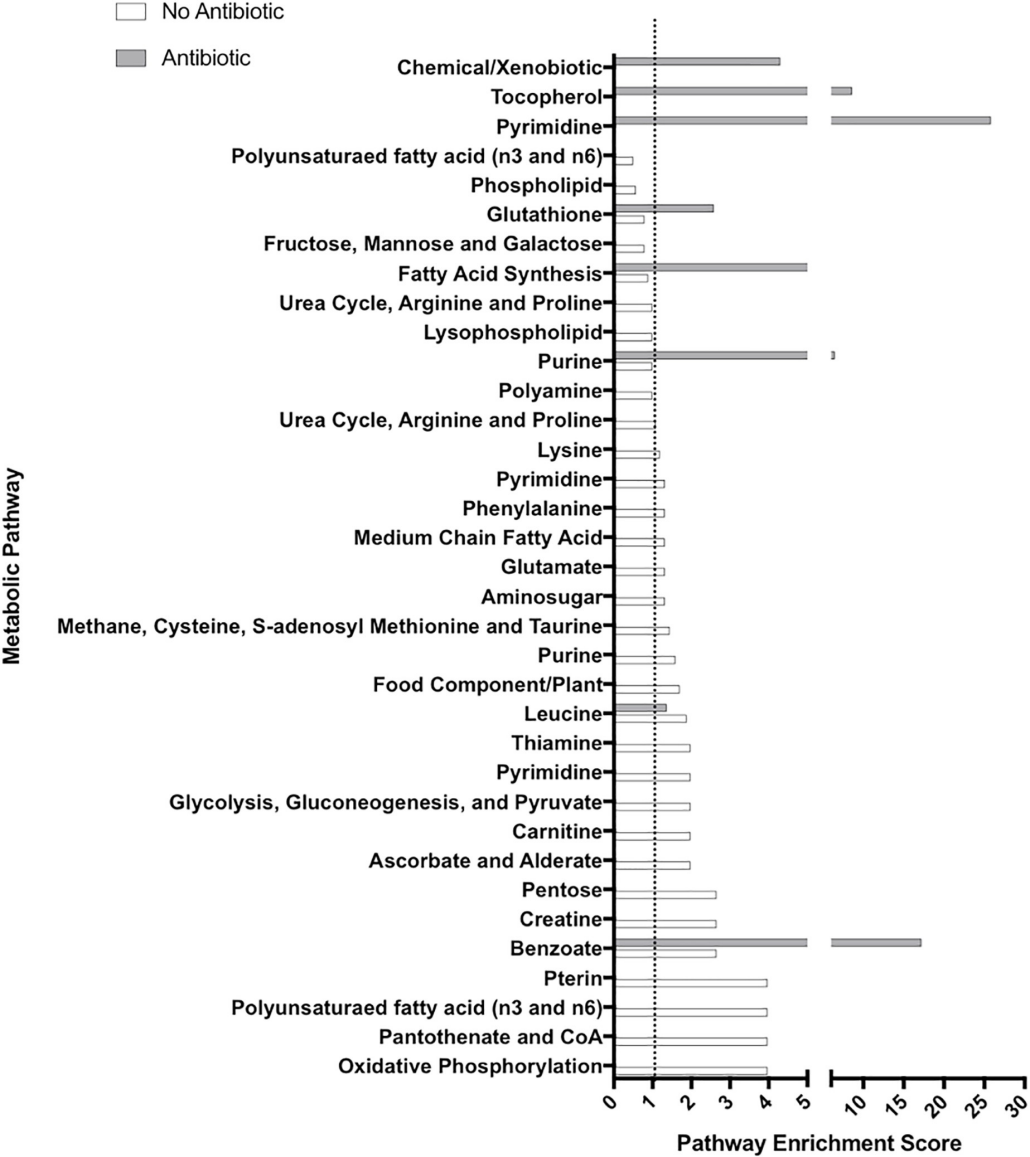
**Pathway enrichment scores were calculated for 100 mmHg versus 5.9 mmHg oxygen in both the presence of antibiotic (grey) and absence of antibiotic (white).** Bars that extend past the dotted line (at a score of 1) were considered the primary pathways contributing to treatment differences. Lack of a white or grey line for any pathway indicates that no metabolites were statistically-different between treatments (p<0.05) within that pathway.

The analysis of tissue culture supernatants indicates the presence of microbially derived metabolites, including lipids, amino acids, and carbohydrates. These metabolites included trimethylamine N-oxide (TMAO), indole-containing metabolites (e.g. indolacetate), and phenyllactate, amongst others. Additionally, these three metabolites were decreased in response to antibiotic treatment (S1 Table). Pathway enrichment scores were calculated to identify pathways that had highest contributions to the treatment differences. In each pathway, a score greater than or equal to 1 was defined as a major contributing pathway [32]. Pathway enrichment scores are displayed for 100 mmHg versus 5.9 mmHg oxygen in the presence and absence of antibiotic (Fig 5). In the presence of antibiotic, 8 pathways distinguished 100 mmHg oxygen from 5.9 mmHg oxygen treatments, while in the absence of antibiotic, 25 pathways distinguished 100 mmHg oxygen from 5.9 mmHg oxygen treatments (Fig 5).

### Antibiotic and oxygen impact epithelial proliferation

**Fig 6:**
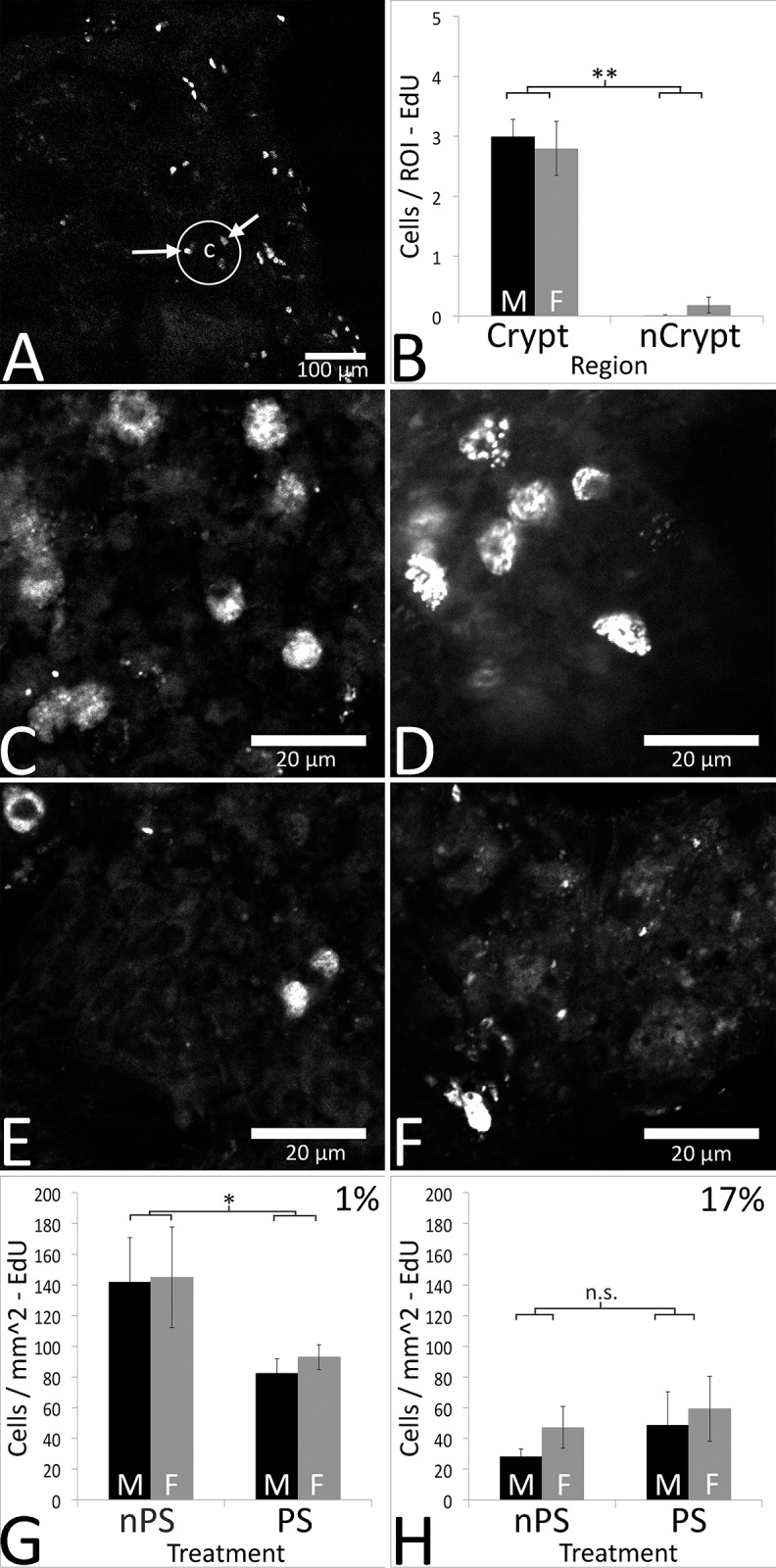
Incorporation of Ethynyl deoxyuridine (EdU) as indicative of DNA synthesis was observed in all oxygen and +/- PS treatments and across all time points (0 – 72h ex vivo). EdU was localized to cells in the colonic crypts (A). This localization was quantified (B), with significantly more EdU^+^ cells per ROI observed in the crypts compared to non-Crypt (nCrypt) regions, regardless of sex (p < 0.05). Representative images of cells in slices from a male participants biopsy show increased EdU^+^ cells in 1% nPS (C) compared to 1% PS (D), both of which are have higher cell counts than 17% nPS (E) and 17% PS (F). Antibiotic had a significant impact on EdU incorporation in slices incubated at 1% oxygen independent of sex (G), with more cells / mm^2^ of tissue seen in nPS slices compared to PS slices. Additionally, oxygen concentration significantly impacted EdU incorporation, with more cells / mm^2^ in 1% slices compared to 17% oxygen cultured slices (G and H). There was no significant impact of PS treatment on slices cultured in 17% oxygen, regardless of sex (H). In B, G and H, black bars are males (M), and grey bars are females (F). Arrows in A point toward stereotypic EdU^+^ cells. “c” represents a colonic crypt, and the circle is encapsulating one crypt. ** Signifies p < 0.01, * signifies p < 0.05, and n.s. = not significant. Data in B, G and H are +/- SEM, n = 5 female, 4 male participants for all EdU experiments except Crypt vs nCrypt analysis, in which n = 3 female, 3 male participants.

Following the finding that antibiotics and oxygen impacted microbial metabolites, mucosal epithelial proliferation was assessed by EdU incorporation in response to antibiotic treatment and oxygen concentration. After 48h ex vivo, slices maintained at 5.9 mmHg oxygen showed significantly more EdU labeling compared to ambient oxygen slices [Fig 6G and 6H; F[(1,14) = 41.1, p < 0.01], regardless of sex [p > 0.5]. Interestingly, antibiotic treatment impacted EdU incorporation in slices maintained at 5.9 mmHg oxygen (Fig 6C and 6D), but not 100 mmHg (Fig 6E and 6F). There was a statistically significant interaction between antibiotic and oxygen conditions [F(1,14) = 10.4, p < 0.01] that was due to the enhanced impact of antibiotic on EdU incorporation at 5.9 mmHg oxygen (Fig 6G and 6H). Mean cell counts within regions of interest (ROIs; circle in Fig 6A) inside colonic crypts were compared to ROIs outside colonic crypts. Significantly more cells incorporated EdU in Crypt regions compared to nCrypt regions, independent of sex [Fig 6B; F(1,12) = 102.9, p < 0.01] and consistent with DNA synthesis being localized primarily to epithelial cells in the crypt proliferative regions.

## Discussion

The current study validates an organotypic slice model for the study of human intestinal physiology ex vivo. Three-dimensional tissue integrity was maintained by embedding the tissue in a solid agarose support that allowed the culture of the diverse cells of the intestine in a physiologically appropriate arrangement. Tissues were maintained for up to 3 days ex vivo, preserving crypt structure, normal cell proliferation and death rates, enteric glia and neuronal fibers, and an immune response to pathogen. Tissue culture supernatants contained known microbial metabolites [9] indicating the maintenance of functional microbiota. These metabolites were sensitive to treatment with antibiotic (PS), demonstrating the influence of antibiotic on microbial populations and/or their metabolite secretory competence. The model further demonstrated a difference in human colonic epithelial health in response to oxygen condition and antibiotic exposure, both known to alter gut microbial diversity [21]. Of paramount importance was that the influence of antibiotic on epithelial proliferation was dependent on oxygen culture condition, further implicating microbes as key influencers of mucosal epithelial biology. These findings highlight the need for better definition of the microbial composition (or the functional metabolite profile output) in ex vivo systems moving forward.

The efficacy of the organotypic model in the present study to elicit a functional immune response ex vivo was validated via a demonstrated T-lymphocyte response to challenge with *S*. *ser*. Typhimurium-GFP. T-lymphocytes in colon biopsy slices showed a more than 2-fold increase in response to pathogen when compared to vehicle (no pathogen, Fig 4F). The increased T lymphocyte count was similar to that seen in mice [33,34], and chickens [13] where T-lymphocytes were up-regulated after dosing with *S*. Typhimurium. This immune response to pathogen ex vivo supports the utility of this model for future studies investigating host pathogen defense and gut mucosal protection in human intestines.

Analysis of colon tissue culture supernatants revealed the presence of a number of microbial metabolites [23] including phenyl containing organic acids (e.g., phenyllactate), the choline/carnitine bacterial breakdown product TMAO, and indole containing metabolites involved in the bacterial metabolism of tryptophan. The substantial impact of both oxygen concentration and antibiotics on the production of metabolites was notable for lipids and amino acids spanning diverse metabolic pathways (Table 1). Interestingly, differences in the influence of oxygen on metabolites were only visible in antibiotic free tissue supernatants. Treatment with penicillin has been shown to decrease bacterial sequence counts and microbial diversity in the cecum of chickens [35], potentially due to antibiotic treatment (PS) resulting in a decrease in select PS-sensitive microbiota, allowing PS-resistant bacteria to flourish. Penicillin treatment has also been shown to directly decrease the microbially derived metabolite phenyllactate, a change also observed in the present study (S1 Table). Similarly, as intermittent hypoxia has been shown to alter gut microbial diversity of mice [36] it is likely that lower oxygen concentrations facilitate the growth of some facultative and anaerobic bacteria, but perhaps decrease the growth rates of aerobic bacteria. A determination of bacterial species that contribute to these metabolic changes will require future metagenomics analyses.

Maintenance of tissue in ambient oxygen conditions has been standard in mammalian cell and tissue culture for decades [37]. While gut cell cultures can survive under these conditions, they do not represent physiologically relevant oxygen concentrations. Oxygen concentrations in the colonic mucosa range from roughly 1% (~6 mmHg) near the lumen, to between 5–10% (~25–70 mmHg) near the vascularized submucosa or deeper muscle layers [38]. The gut oxygen gradient is important for regulating the transcription factor hypoxia inducible factor-1 alpha (HIF-1α) that helps control numerous metabolic and physiologic pathways, including creatine metabolism [39] and intestinal angiogenesis [40]. Recapitulating this oxygen gradient ex vivo is a difficult task, and one that was not attempted in the current study. The present study mimicked the in vivo oxygen concentration seen in the apical colonic mucosa (approximately 1%, ~6 mmHg), ex vivo. Under 5.9 mmHg oxygen conditions, significantly more crypt cells underwent DNA synthesis, marked by EdU incorporation, when compared to slices cultured in ambient (100 mmHg) oxygen. In addition, the exclusion of penicillin-streptomycin treatment, and subsequent culture in 5.9 mmHg oxygen lead to a further significant increase in EdU incorporation compared to tissue cultured without PS. The amount of EdU incorporation observed at 5.9 mmHg oxygen without PS is consistent with the rates of another thymidine analog, 5-bromo-2’-deoxyuridine, incorporation seen in numerous in vivo human colonic mucosal proliferation studies [31,41]. The statistical interaction between oxygen and PS conditions pointed towards the importance of oxygen tension for antibiotics to have an effect on DNA synthesis in colonic mucosal crypts ex vivo. Altered oxygen availability can lead to varied bacterial metabolism [42] and secretion of products such as virulence factors [43]. The lowered oxygen concentration in the current study could thus influence microbial metabolism, potentially rendering bacteria more susceptible to antibiotic treatment. Further investigation into the interaction between oxygen concentration in culture and antibiotics influence on microbial metabolites is warranted.

## Conclusions

This report provides an organotypic slice model for human intestinal tissues ex vivo that optimizes cellular diversity and 3-dimensional integrity. This model can be used to tease apart complex multi–system–gut interactions with translational potential, including enteric pathogen interactions and intestinal immune responses to host—microbiome interactions. Importantly, cell proliferation in this model was impacted by two factors that influence microbial diversity and function: oxygen tension and antibiotic exposure. The physiological relevance of these factors may be missed under standard culture conditions with higher oxygen tension and exogenous antibiotic added, especially in the context of how commensal microbial interactions impact tissue function. The current findings set the stage for use of the present organotypic slice model for studies of the complex roles of commensal microbiota and microbially-derived metabolite secretions in regulating human gut health. There is significant cell diversity in the colon along with unique physiologic oxygen tensions that are coupled with the presence of differentially active tissue and microbial metabolism. The system described in the current study offers important similarities to the intestinal wall ex vivo. It provides for live microscopic imaging of fluorescently tagged bacteria interacting with the native human gut environment, setting the stage for unravelling specific microbial–host cell interactions. For example, the complexity of local immune cell responses to pathogen will be more accessible in vitro as in the T-cell proliferation observed in the current study. Particular microbial metabolites may be manipulated to discern potential cellular mechanisms that may be disturbed in different disease states or tested for potential therapeutic value.

## Supporting information

S1 Table Supernatant metabolite abundance fold differences when comparing across oxygen concentrations and in the presence or absence of antibiotic. (PDF)

## References

[1] National Institutes of Health, U.S. Department of Health and Human Services. Opportunities and Challenges in Digestive Diseases Research: Recommendations of the National Commission on Digestive Diseases. Bethesda, MD: National Institutes of Health; 2009 NIH Publication 08–6514.

[2] Adams SM, Bornemann PH. Ulcerative Colitis. Am Fam Physician. 2013;87: 699–705. 23939448

[3] Autrup H, Barrett LA, Jackson FE, Jesudason ML, Stoner G, Phelps P, Trump BF, Harris CC. Explant culture of human colon. Gastroenterology. 1978;74:1248–1257. 648817

[4] Liu YA, Chung YC, Pan ST, Shen MY, Hou YC, Peng SJ, et al 3-D imaging, illustration, and quantitation of enteric glial network in transparent human colon mucosa. Neurogastroenterol Motil. 2013;25:e324–e338. 10.1111/nmo.12115 23495930

[5] Yu YB, Li YQ. Enteric glial cells and their role in the intestinal epithelial barrier. World J Gastroenterol. 2014;20:11273–11280. 10.3748/wjg.v20.i32.11273 25170211 PMC4145765

[6] Sharkey KA. Emerging roles for enteric glia in gastrointestinal disorders. J Clin Invest. 2015;125:918–925. 10.1172/JCI76303 25689252 PMC4362226

[7] Pochard C, Coquenlorge S, Freyssinet M, Naveilhan P, Bourreille A, Neunlist M, et al The multiple faces of inflammatory enteric glial cells: is Crohn’s disease a gliopathy? Am J Physiol Gastrointest Liver Physiol. 2018; 10.1152/ajpgi.00016 29517926

[8] Lee W, Hase K. Gut microbiota-generated metabolites in animal health and disease. Nat Chem Biol. 2014;10:416–424. 10.1038/nchembio.1535 24838170

[9] Brown JM, Hazen SL. Targeting of microbe-derived metabolites to improve human health: The next frontier for drug discovery. J. Biol. Chem. 2017;292:8560–8568. 10.1074/jbc.R116.765388 28389555 PMC5448085

[10] Louis P, Hold GL, Flint HJ. The gut microbiota, bacterial metabolites and colorectal cancer. Nature Reviews Microbiology; 2014; 10.1038/nrmicro3344 25198138

[11] Berdy J. Bioactive Microbial Metabolites. J. Antibiot. 2005;58:1–26. 10.1038/ja.2005.1 15813176

[12] Jepson MA CM. The role of M cells in Salmonella infection. Microbes Infect. 2001;3:1183–90. 11755406 10.1016/s1286-4579(01)01478-2

[13] Bai SP, Huang Y, Luo YH, Wang LL, Ding XM, Wang JP, et al Alteration in lymphocytes responses, cytokine and chemokine profiles in laying hens infected with Salmonella Typhimurium. Vet Immunol Immunopathol. 2014;160:235–243. 10.1016/j.vetimm.2014.05.015 24986046

[14] Lopez-Medina M, Carrillo-Martin I, Leyva-Rangel J, Alpuche-Aranda C, and Ortiz-Navarrete V. Salmonella impairs CD8 T cell response through PD-1: PD-L axis. Immunobiology. 2015;220:1369–1380. 10.1016/j.imbio.2015.07.005 26210046

[15] Kelly CJ, Colgan SP. Breathless in the Gut: Implications of Luminal O2 for Microbial Pathogenicity. Cell Host Microbe. 2016;19:427–428. 10.1016/j.chom.2016.03.014 27078062 PMC5256639

[16] Thermann M, Jostarndt L, Eberhard F, Richter H, Sass W. Oxygen supply of the human small intestine in mechanical ileus. Langenbecks Arch Chir. 1985;363:179–84. 3921788 10.1007/BF01261291

[17] Albenberg L, Esipova TV, Judge CP, Bittinger K, Chen J, Laughlin A, et al Correlation Between Intraluminal Oxygen Gradient and Radial Partitioning of Intestinal Microbiota. Gastroenterology. 2014;147:1055–1063. 10.1053/j.gastro.2014.07.020 25046162 PMC4252572

[18] von Martels JZH, Sadabad MS, Bourgonje AR, Blokzijl T, Dijkstra G, Faber KN, et al The role of gut microbiota in health and disease: In vitro modeling of host-microbe interactions at the aerobe-anaerobe interphase of the human gut. Anaerobe. 2017;44:3–12. 10.1016/j.anaerobe.2017.01.001 28062270

[19] Schwerdtfeger LA, Ryan EP, and Tobet SA. An organotypic slice model for ex vivo study of neural, immune, and microbial interactions of mouse intestine. Am J Physiol Gastrointest Liver Physiol. 2016;310:G240–248. 10.1152/ajpgi.00299.2015 26680736 PMC4754739

[20] Chowdhury SR, King DE, Willing BP, Band MR, Beever JE, Lane AB, et al Transcriptome profiling of the small intes- tinal epithelium in germfree versus conventional piglets. BMC Genomics 2007;8:215 10.1186/1471-2164-8-215 17615075 PMC1949829

[21] Tulstrup MV, Christensen EG, Carvalho V, Linninge C, Ahrne S, Hojberg O, et al Antibiotic Treatment Affects Intestinal Permeability and Gut Microbial Composition in Wistar Rats Dependent on Antibiotic Class. PloS One. 2015;10: e0144854 10.1371/journal.pone.0144854 26691591 PMC4686753

[22] Garner CD, Antonopoulos DA, Wagner B, Duhamel GE, Keresztes I, Ross DA, et al Perturbation of the small intestine microbial ecology by streptomycin alters pathology in a Salmonella enterica serovar typhimurium murine model of infection. Infect Immun. 2009;77:2691–2702. 10.1128/IAI.01570-08 19433544 PMC2708583

[23] Sun J, Schnackenberg LK, Khare S, Yang X, Greenhaw J, Salminen W, et al Evaluating effects of penicillin treatment on the metabolome of rats. J Chromatogr B. 2013;932:134–143. 10.1016/j.jchromb.2013.05.030 23831706

[24] Gustafsson JK, Ermund A, Johansson ME, Schutte A, Hansson GC, and Sjovall H. An ex vivo method for studying mucus formation, properties, and thickness in human colonic biopsies and mouse small and large intestinal explants. Am J Physiol Gastrointest Liver Physiol. 2012;302:G430–438. 10.1152/ajpgi.00405.2011 22159279 PMC4073982

[25] Mapes B, Chase M, Hong E, Ludvik A, Ceryes K, Huang Y, et al Ex vivo culture of primary human colonic tissue for studying transcriptional responses to 1alpha,25(OH)2 and 25(OH) vitamin D. Physiol Genomics. 2014;46:302–308. 10.1152/physiolgenomics.00194.2013 24550213 PMC4035659

[26] Drew JE, Farquharson AJ, Vase H, Carey FA, Steele RJ, Ross RA, et al Molecular Profiling of Multiplexed Gene Markers to Assess Viability of Ex Vivo Human Colon Explant Cultures. Biores Open Access. 2015;4:425–430. 10.1089/biores.2015.0029 26634188 PMC4652222

[27] Yissachar N, Zhou Y, Ung L, Lai NY, Mohan JF, Ehrlicher A, et al An Intestinal Organ Culture System Uncovers a Role for the Nervous System in Microbe-Immune Crosstalk. Cell. 2017;168:1135–1148. 10.1016/j.cell.2017.02.009 28262351 PMC5396461

[28] Shah P, Fritz JV, Glaab E, Desai MS, Greenhalgh K, Frachet A, et al A microfluidics-based in vitro model of the gastrointestinal human-microbe interface. Nat Commun. 2016;7: 11535 10.1038/ncomms11535 27168102 PMC4865890

[29] Kumar A HA, Forster GM, Goodyear AW, Weir TL, Leach JE, Dow SW, et al Dietary rice bran promotes resistance to Salmonella enterica serovar Typhimurium colonization in mice. BMC Microbiol. 2012;12:71–80. 10.1186/1471-2180-12-71 22583915 PMC3390288

[30] Nealon NJ, Yuan L, Yang X, Ryan EP. Rice Bran and Probiotics Alter the Porcine Large Intestine and Serum Metabolites for Protection against Human Rotavirus Diarrhea. Front Microbiol. 2017;8:653 10.3389/fmicb.2017.00653 28484432 PMC5399067

[31] Potten CS KM, Roberts SA, Rew DA, Wilson GD. Measurement of in vivo proliferation in human colorectal mucosa using bromodeoxyuridine. Gut. 1992;33:71–78. 10.1136/gut.33.1.71 1740282 PMC1373868

[32] Nealon NJ, Worcester CR, and Ryan EP. Lactobacillus paracasei metabolism of rice bran reveals metabolome associated with Salmonella Typhimurium growth reduction. J Appl Microbiol. 2017;122:1639–1656. 10.1111/jam.13459 28371001 PMC5518229

[33] Li X, Yao Y, Wang X, Zhen Y, Thacker PA, Wang L, et al Chicken egg yolk antibodies (IgY) modulate the intestinal mucosal immune response in a mouse model of Salmonella typhimurium infection. Int Immunopharmacol. 2016;36:305–314. 10.1016/j.intimp.2016.04.036 27214338 PMC7106048

[34] Rydstrom A & Wick MJ. Monocyte Recruitment, Activation, and Function in the Gut-Associated Lymphoid Tissue during Oral Salmonella Infection. J Immunol. 2007;178:5789–5801. 17442963 10.4049/jimmunol.178.9.5789

[35] Singh P, Karimi A, Devendra K, Waldroup PW, Cho KK, Kwon YM. Influence of penicillin on microbial diversity of the cecal microbiota in broiler chickens. Poult Sci. 2013;92:272–276. 10.3382/ps.2012-02603 23243258

[36] Moreno-Indias I, Torres M, Montserrat JM, Sanchez-Alcoholado L, Cardona F, Tinahones FJ, et al Intermittent hypoxia alters gut microbiota diversity in a mouse model of sleep apnoea. Eur Respir J. 2015;45:1055–1065. 10.1183/09031936.00184314 25537565

[37] Rodriguez-Hernandez CO MA, Gonzalez FJA, Guerrero-Barrera AL. Cell culture: History, Development and Prospects. Int J Curr Res Aca Rev. 2014;2:188–200.

[38] Zheng L, Kelly CJ, and Colgan SP. Physiologic hypoxia and oxygen homeostasis in the healthy intestine. A Review in the Theme: Cellular Responses to Hypoxia. American J Physiol Cell Physiol. 2015;309:C350–360. 10.1152/ajpcell.00191.2015 PMC4572369 26179603

[39] Glover LE, Bowers BE, Saeedi B, Ehrentraut SF, Campbell EL, Bayless AJ, et al Control of creatine metabolism by HIF is an endogenous mechanism of barrier regulation in colitis. Proc Natl Acad Sci U S A. 2013;110:19820–19825. 10.1073/pnas.1302840110 24248342 PMC3856803

[40] Bakirtzi K, West G, Fiocchi C, Law IKM, Iliopoulos D, Pothoulakis C. The Neurotensin-HIF-1α-VEGFα Axis Orchestrates Hypoxia, Colonic Inflammation, and Intestinal Angiogenesis. Am J Pathol. 2014;184:3405–3414. 10.1016/j.ajpath.2014.08.015 25307345 PMC4258496

[41] Khan S, Raza A, Petrelli N, Mittleman A. In Vivo Determinations of Labelling Index of Metastatic Colorectal Carcinoma and Normal Colonic Mucosa Using Intravenous Infusions of Bromodeoxyuridine. J Surg Oncol. 1988;39:114–118. 3172791 10.1002/jso.2930390209

[42] Wessel AK, Arshad TA, Fitzpatrick M, Connell JL, Bonnecaze RT, Shear JB, et al Oxygen Limitation within a Bacterial Aggregate. mBio. 2014;5:e00992–14. 10.1128/mBio.00992-14 24736225 PMC3994514

[43] Schertzer JW, Brown SA, Whiteley M. Oxygen levels rapidly modulate Pseudomonas aeruginosa social behaviors via substrate limitation of PqsH. Mol Microbiol. 2010;77(6):1527–1538. 10.1111/j.1365-2958.2010.07303.x 20662781 PMC3098721

