# Supplementary material for: Human colon function ex vivo: Dependence on oxygen and sensitivity to antibiotic
Source: PLoS One. 2019 May 16;14(5):e0217170. doi: 10.1371/journal.pone.0217170 (PMC6522050; doi:10.1371/journal.pone.0217170)
Supplement: S1 Table — (PDF) [file pone.0217170.s001.pdf]

S1 Table

	Database[1]			Fold Difference[2,3]	
Biochemical Name	KEGG	PUBCHEM	HMDB	PS/NPS[4]	20%/1%[5]
(N(1) + N(8))-acetylspermidine	-	-	-	**0.0422**	1.2451
1-(1-enyl-stearoyl)-GPE (P-18:0)	-	-	-	1.1576	1.038
1-methyl-4-imidazoleacetate	C05828	75810	HMDB02820	0.9844	**1.6007**
1-methylnicotinamide	C02918	10129985	HMDB00699	**2.0959**	1.2147
1-oleoyl-glycerophospho-ethanolamine (GPE; 18:1)	-	9547071	HMDB11506	**0.2051**	1.0769
1-oleoyl-glycerophospho-glycine (GPG; 18:1)	-	-	-	**0.351**	0.9944
1-palmitoyl-2-oleoyl-glycerophospho-choline (GPC; 16:0/18:1)	-	6436017	HMDB07972	0.9525	0.9488
1-palmitoyl-2-oleoyl-GPE (16:0/18:1)	-	5283496	HMDB05320	**0.4211**	1.0898
1-palmitoyl-GPE (16:0)	-	9547069	HMDB11503	**0.1138**	1.2431
1-palmitoyl-GPG (16:0)	-	3300276	-	**0.1486**	1.1921
1-stearoyl-GPC (18:0)	-	497299	HMDB10384	1.1167	1.0997
1-stearoyl-GPE (18:0)	-	9547068	HMDB11130	**0.7486**	1.0686
1-stearoyl-glycerophospho-inositol (GPI; 18:0)	-	-	HMDB61696	1.31	1.1588
2,3-dihydroxy-2-methylbutyrate	-	301941	HMDB29576	**0.0796**	1.1258
2,3-dihydroxyisovalerate	C04039	677	HMDB12141	**0.0203**	1.0238
2,4-dichlorophenol sulfate	-	9837697	-	***2.2402***	***0.2024***
2-aminoadipate	C00956	469	HMDB00510	**0.3218**	0.8916
2'-deoxycytidine	C00881	13711	HMDB00014	**2.3968**	0.8279
2'-deoxyuridine	C00526	13712	HMDB00012	**2.6871**	1.1013
2-hydroxy-3-methylvalerate	-	164623	HMDB00317	***0.0056***	*0.6062*
2-hydroxyadipate	C02360	193530	HMDB00321	**0.1444**	0.7765
2-hydroxybutyrate/2-hydroxyisobutyrate	-	-	-	***0.341***	***0.4398***
2-hydroxyglutarate	C02630	43	HMDB00606	**0.0091**	1.1134
2-methylcitrate/homocitrate	-	-	-	**0.1082**	2.7146
2-oxoadipate	C00322	71	HMDB00225	**0.0108**	3.1639
2-oxoarginine	C03771	558	HMDB04225	**0.4882**	1.2694
3-(4-hydroxyphenyl)lactate	C03672	9378	HMDB00755	**0.0128**	1.2101
3-deoxyoctulosonate	-	4636210	-	**0.0771**	1.3928
3-hydroxy-3-methylglutarate	C03761	1662	HMDB00355	1.3374	1.7768
3-hydroxybutyrate (BHBA)	C01089	441	HMDB00357	0.5105	1.1556
3-hydroxydecanoate		26612	HMDB02203	**0.2259**	1.4838
3-hydroxyisobutyrate	C06001	87	HMDB00336	0.8546	**4.713**
3-hydroxylaurate	-	94216	HMDB00387	**0.134**	1.8042
3-hydroxymyristate	-	16064	-	**0.2848**	1.3447
3-hydroxyoctanoate	-	26613	HMDB01954	**0.1609**	1.3401
3-methyl-2-oxobutyrate	C00141	49	HMDB00019	0.8698	1.5135
3-methyl-2-oxovalerate	C00671	47	HMDB03736	**0.2412**	1.4748
3-sulfo-L-alanine	C00506	72886	HMDB02757	**6.3592**	1.1185
3-ureidoisobutyrate	C05100	160663	HMDB02031	**0.4537**	1.2057
3-ureidopropionate	C02642	111	HMDB00026	**0.347**	0.9643
4-acetamidobenzoate	D03836	19266	-	**0.283**	1.0645
4-acetamidobutanoate	C02946	18189	HMDB03681	**0.3559**	**4.3372**
4-ethylphenylsulfate	C13637	-	-	***2.7144***	***0.5132***

4-guanidinobutanoate	C01035	500	HMDB03464	**0.4857**	1.0852
4-hydroxyphenylpyruvate	C01179	979	HMDB00707	**0.0256**	1.4647
4-imidazoleacetate	C02835	96215	HMDB02024	**0.1118**	1.4199
4-methyl-2-oxopentanoate	C00233	70	HMDB00695	0.4224	1.4294
4-methylbenzenesulfonate	C06677	6101	-	0.5982	0.5664
5-(2-Hydroxyethyl)-4-methylthiazole	C04294	1136	-	**0.7255**	1.1441
5-aminovalerate	C00431	138	HMDB03355	***0.0822***	*0.6266*
5-hydroxylysine	C16741	1029	HMDB00450	**1.3772**	0.838
5-methylthioadenosine	C00170	439176	HMDB01173	**0.4323**	0.7994
5-oxoproline	C01879	7405	HMDB00267	*1.2909*	***5.1653***
6-oxopiperidine-2-carboxylate	-	3014237	HMDB61705	**0.0073**	1.8124
7-methylguanine	C02242	11361	HMDB00897	**0.0541**	1.1447
acetylcarnitine (C2)	C02571	1	HMDB00201	**4.9199**	1.4237
aconitate [cis or trans]	-	-	-	**0.0582**	1.4204
adenine	C00147	190	HMDB00034	**0.1767**	1.0285
agmatine	C00179	199	HMDB01432	**0.1028**	0.6998
alanine	C00041	5950	HMDB00161	**6.6736**	0.8385
allantoin	C02350	204	HMDB00462	**2.2118**	0.676
alpha-hydroxyisocaproate	C03264	83697	HMDB00746	**0.0026**	0.608
alpha-hydroxyisovalerate	-	99823	HMDB00407	**0.0046**	0.95
alpha-ketoglutaramate	-	-	-	**0.4266**	**1.5975**
alpha-ketoglutarate	C00026	51	HMDB00208	**0.0329**	2.0285
alpha-tocopherol	C02477	14985	HMDB01893	**0.5291**	1.1258
alpha-tocopherol acetate	C13202	2117	HMDB34227	0.87	1.0924
arabonate/xylonate	-	-	-	***1.9475***	***1.5669***
arachidonate (20:4n6)	C00219	444899	HMDB01043	1.273	**0.5141**
arginine	C00062	232	HMDB00517	**22.8578**	0.859
asparagine	C00152	6267	HMDB00168	**183.3345**	1.2319
aspartate	C00049	5960	HMDB00191	**6.7202**	0.7734
benzoate	C00180	243	HMDB01870	0.8253	0.8114
beta-hydroxyisovalerate	-	69362	HMDB00754	1.0793	1.4263
betaine	C00719	247	HMDB00043	1.6123	2.4538
biotin	C00120	171548	HMDB00030	**0.8786**	1.0531
cadaverine	C01672	273	HMDB02322	***0.0201***	*0.4265*
caproate (6:0)	C01585	8892	HMDB00535	0.7097	1.6797
caprylate (8:0)	C06423	379	HMDB00482	**0.4616**	1.0633
carnitine	C00318	10917	HMDB00062	**5.1771**	1.8015
choline	C00114	305	HMDB00097	**1.3477**	1.1243
choline phosphate	C00588	1014	HMDB01565	**0.7326**	1.025
cis-urocanate	-	1549103	HMDB34174	**0.2067**	1.0344
citrate	C00158	311	HMDB00094	**0.1459**	**3.0763**
citrulline	C00327	9750	HMDB00904	1.4624	0.9913
creatine	C00300	586	HMDB00064	0.7904	1.7461
creatinine	C00791	588	HMDB00562	**2.5136**	0.7693
cysteine	C00097	5862	HMDB00574	**3.1885**	1.1769
cysteine s-sulfate	C05824	115015	HMDB00731	**160.7614**	2.2195
cysteine sulfinic acid	C00606	109	HMDB00996	**3.6761**	0.9508
cysteine-glutathione disulfide	-	4247235	HMDB00656	**2.2084**	**2.4116**
cysteinylglycine disulfide	-	-	HMDB00709	**6.3234**	1.468

cystine	C00491	67678	HMDB00192	**21.0063**	1.3741
cytidine	C00475	6175	HMDB00089	***5.3697***	*1.3168*
cytosine	C00380	597	HMDB00630	0.5698	1.4199
deoxycarnitine	C01181	134	HMDB01161	***0.0057***	*1.1325*
diacetylspermidine	-	-	-	**0.1923**	1.151
dihomo-linoleate (20:2n6)	C16525	6439848	HMDB05060	*0.9362*	***0.4834***
dihomo-linolenate (20:3n3 or n6)	C03242	5280581	HMDB02925	*0.9991*	***0.4844***
dihydroorotate	C00337	648	HMDB03349	***0.209***	***0.3***
dimethylarginine (Symmetric and Assymetric)	C03626	123831	HMDB01539	1.0663	1.0362
dodecadienoate (12:2)	-	-	-	***0.398***	***2.1497***
eicosapentaenoate (EPA; 20:5n3)	C06428	446284	HMDB01999	***1.4639***	***0.6807***
erucate (22:1n9)	C08316	5281116	HMDB02068	**1.2439**	1.0827
erythritol	C00503	222285	HMDB02994	**0.2976**	1.4114
erythronate	-	2781043	HMDB00613	0.8711	1.3089
folate	C00504	6037	HMDB00121	**0.8888**	1.0803
formiminoglutamate	C00439	439233	HMDB00854	**0.0702**	0.9958
fructose	C00095	5984	HMDB00660	**20.9323**	1.2444
fumarate	C00122	444972	HMDB00134	**0.0882**	0.4654
gamma-glutamylmethionine	-	7009567	HMDB29155	1.4332	1.5369
gamma-glutamylthreonine	-	76078708	HMDB29159	1.3979	2.6691
gluconate	C00257	10690	HMDB00625	0.7856	1.8157
glucose	C00031	79025	HMDB00122	**808.2145**	4.3005
glucuronate	C00191	444791	HMDB00127	**1.5971**	1.1658
glutamate	C00025	611	HMDB00148	**2.4599**	1.9824
glutamine	C00064	5961	HMDB00641	**3.921**	1.0715
glutarate (C5-DC)	C00489	743	HMDB00661	**0.0072**	2.0572
glycerate	C00258	752	HMDB00139	**0.3938**	0.9887
glycerol	C00116	753	HMDB00131	***3.4654***	***0.4221***
glycerol 3-phosphate	C00093	754	HMDB00126	**0.0921**	0.8528
glycerophosphoethanolamine	C01233	123874	HMDB00114	**1.948**	0.8676
glycerophosphoglycerol	C03274	439964	-	1.8645	0.969
glycerophosphorylcholine	C00670	71920	HMDB00086	**4.5688**	0.8988
glycerophosphoserine	-	3081457	-	**2.4353**	0.8236
glycine	C00037	750	HMDB00123	**58.0259**	1.2767
guanidine	C17349	3520	HMDB01842	0.9316	0.9403
guanidinoacetate	C00581	763	HMDB00128	0.8315	1.79
guanine	C00242	764	HMDB00132	**0.1639**	1.724
guanosine	C00387	6802	HMDB00133	0.818	**1.2991**
guanosine 5'-triphosphate	C00044	6830	HMDB01273	1.0562	1.0217
HEPES	-	23831	-	**0.9622**	**1.0434**
heptanoate (7:0)	C17714	8094	HMDB00666	1.0934	1.0476
histamine	C00388	774	HMDB00870	0.65	0.637
histidine	C00135	6274	HMDB00177	8.3691	0.6545
hypoxanthine	C00262	790	HMDB00157	1.3015	1.1167
imidazole lactate	C05568	440129	HMDB02320	**0.0033**	1.1903
imidazole propionate	-	70630	HMDB02271	**0.0337**	0.6602
indole-3-carboxylic acid	C19837	69867	HMDB03320	**0.4221**	**1.4947**
indoleacetate	C00954	802	HMDB00197	**0.0981**	1.2664
indolelactate	C02043	92904	HMDB00671	**0.008**	0.9858
inosine	C00294	6021	HMDB00195	**0.5046**	1.5612

isobutyrylglycine	-	10855600	HMDB00730	**0.6484**	**0.6231**
isocitric lactone	-	98259	-	***0.3342***	***2.2191***
isoleucine	C00407	6306	HMDB00172	1.9351	0.483
isovalerate (i5:0)	C08262	10430	HMDB00718	**0.0818**	**2.7074**
isovalerylglycine	-	546304	HMDB00678	*0.9558*	***0.6477***
kynurenate	C01717	3845	HMDB00715	**0.0591**	1.3584
kynurenine	C00328	161166	HMDB00684	**2.0904**	1.3017
lactate	C00186	612	HMDB00190	1.2289	**0.2069**
leucine	C00123	6106	HMDB00687	**2.5884**	0.4865
linoleate (18:2n6)	C01595	5280450	HMDB00673	**2.166**	**0.5977**
linolenate [alpha or gamma; (18:3n3 or 6)]	C06426	5280934	HMDB03073	**3.0045**	0.559
lysine	C00047	5962	HMDB00182	**42.4422**	1.1307
malate	C00149	525	HMDB00156	**0.1668**	0.4581
malonate	C00383	867	HMDB00691	**0.2733**	1.2525
malonylcarnitine	-	22833583	HMDB02095	0.9662	1.1769
maltol	C11918	8369	HMDB30776	**44.2799**	1.376
maltose	C00208	10991489	HMDB00163	**2.2192**	1.2926
mannose	C00159	18950	HMDB00169	**1.6325**	1.1114
methionine	C00073	6137	HMDB00696	***6.3779***	***0.4728***
methionine sulfoxide	C02989	158980	HMDB02005	**8.0633**	0.4732
methyl indole-3-acetate	-	74706	HMDB29738	**0.0962**	**2.3227**
methylsuccinate	-	10349	HMDB01844	**0.5393**	1.1247
3-(N-morpholino)propanesulfonic acid (MOPS)	-	70807;2723950	-	**0.8086**	0.9982
myo-inositol	C00137	892	HMDB00211	1.0398	1.0525
N6,N6,N6-trimethyllysine	C03793	440120	HMDB01325	**0.6144**	0.828
N6-acetyllysine	C02727	92832	HMDB00206	**0.1975**	1.9273
N-acetylalanine	C02847	88064	HMDB00766	**0.254**	1.3441
N-acetylarginine	C02562	67427	HMDB04620	*0.3505*	***0.3073***
N-acetylasparagine	-	99715	HMDB06028	**0.2145**	1.2544
N-acetylaspartate (NAA)	C01042	65065	HMDB00812	**0.071**	1.3962
N-acetylcysteine	C06809	12035	HMDB01890	1.1491	0.9408
N-acetylglucosamine/N-acetylgalactosamine	-	24139	HMDB00215	1.2364	**2.0567**
N-acetylglutamate	C00624	70914	HMDB01138	**0.1379**	1.4232
N-acetylglutamine	C02716	182230	HMDB06029	**0.0626**	1.1265
N-acetylhistamine	C05135	69602	HMDB13253	**0.4822**	1.4336
N-acetylhistidine	C02997	75619	HMDB32055	**0.0804**	1.0363
N-acetylisoleucine	-	2802421	HMDB61684	**0.0269**	1.4932
N-acetyl-isoputreanine	-	-	-	**0.1004**	2.4969
N-acetylleucine	C02710	70912	HMDB11756	**0.0946**	0.9754
N-acetylmethionine	C02712	448580	HMDB11745	***0.051***	*0.5251*
N-acetylmethionine sulfoxide	-	193368	-	**0.1072**	1.0635
N-acetylphenylalanine	C03519	74839	HMDB00512	**0.0762**	1.5321
N-acetylproline	-	322640	-	1.2485	0.9874
N-acetylputrescine	C02714	122356	HMDB02064	**0.0256**	0.8357
N-acetylserine	-	65249	HMDB02931	**0.1682**	1.4827
N-acetylthreonine	-	152204	-	**0.3477**	1.0751
N-acetyltryptophan	C03137	700653	HMDB13713	**0.4651**	1.32
N-acetyltyrosine	-	68310	HMDB00866	**0.2021**	1.2845
N-acetylvaline	-	66789	HMDB11757	**0.0755**	1.637

N-alpha-acetylornithine	C00437	439232	HMDB03357	***0.2797***	***0.2843***
N-delta-acetylornithine	-	9920500	-	**0.1071**	2.7506
N-formylanthranilic acid	C05653	101399	HMDB04089	**0.6683**	0.9248
N-formylmethionine	C03145	439750	HMDB01015	***0.0835***	*0.6744*
N-formylphenylalanine	-	759256	-	***0.2592***	***0.6995***
nicotinamide	C00153	936	HMDB01406	**235.6017**	0.9482
nicotinamide riboside	C03150	439924	HMDB00855	**0.0275**	0.7568
nicotinate	C00253	938	HMDB01488	**0.0804**	1.2255
ornithine	C00077	6262	HMDB03374	***2.6278***	***0.2018***
orotate	C00295	967	HMDB00226	**0.0302**	0.5441
orotidine	-	92751	HMDB00788	**0.3283**	0.9772
o-Tyrosine	-	91482	HMDB06050	0.8098	0.8361
oxalate (ethanedioate)	C00209	971	HMDB02329	***0.5899***	***2.0303***
pantothenate	C00864	6613	HMDB00210	0.9418	1.0783
penicillin G	C05551	5904	HMDB15186	**10.8042**	1.1646
phenol red	C12600	4766	-	1.0701	1.053
phenol sulfate	C02180	74426	HMDB60015	1.5021	0.571
phenylacetate	C07086	999	HMDB00209	**0.1936**	1.7507
phenylacetylglycine	C05598	68144	HMDB00821	**238.3901**	1.0429
phenylalanine	C00079	6140	HMDB00159	**1.6916**	1.1013
phenyllactate	C05607	3848	HMDB00779	**0.0058**	1.1619
phenylpyruvate	C00166	997	HMDB00205	**0.0463**	**2.4754**
phosphate	C00009	1061	HMDB01429	1.0204	0.9847
p-hydroxybenzaldehyde	C00633	126	HMDB11718	**0.139**	**3.0082**
pipecolate	C00408	849	HMDB00070	**0.073**	1.482
prephenic acid	C00254	1028	HMDB12283	**0.1314**	**2.7017**
proline	C00148	145742	HMDB00162	**50.2089**	0.8052
pseudouridine	C02067	15047	HMDB00767	1.3036	1.0915
putrescine	C00134	1045	HMDB01414	*1.691*	***0.3841***
pyridoxal	C00250	1050	HMDB01545	**2.4721**	1.0497
pyridoxamine	C00534	1052	HMDB01431	**1.9509**	1.0787
pyridoxate	C00847	6723	HMDB00017	0.6362	1.5459
pyridoxine (Vitamin B6)	C00314	1054	HMDB02075	**0.0355**	0.7413
pyruvate	C00022	1060	HMDB00243	**4.6099**	1.1908
quinate	C00296	6508	HMDB03072	**0.3856**	0.9122
riboflavin (Vitamin B2)	C00255	493570	HMDB00244	**0.4987**	1.1429
ribonate	C01685	5460677	HMDB00867	**0.713**	1.17
S-1-pyrroline-5-carboxylate	C04322	1196	HMDB01301	1.084	**1.3649**
salicylate	C00805	338	HMDB01895	**0.1278**	1.4384
sebacate (C10-DC)	C08277	5192	HMDB00792	1.1021	0.9804
sedoheptulose	-	5459879	HMDB03219	**0.4701**	1.1118
serine	C00065	5951	HMDB00187	**194.6474**	2.0466
spermidine	C00315	1102	HMDB01257	0.9549	0.9479
streptomycin	-	5999	-	**2.5474**	1.0129
succinate	C00042	1110	HMDB00254	**0.0089**	0.3952
succinimide	C07273	11439	-	**0.367**	1.154
sulfate	C00059	1118	HMDB01448	**3.8232**	1.0547
thiamin (Vitamin B1)	C00378	1130	HMDB00235	***0.304***	*0.5615*
thioproline	-	93176	-	**1.8682**	1.2537

threonate	C01620	151152	HMDB00943	**0.4656**	1.5783
threonine	C00188	6288	HMDB00167	**132.011**	1.6174
thymidine	C00214	5789	HMDB00273	*1.198*	*1.3833*
thymine	C00178	1135	HMDB00262	**0.0351**	0.9375
trans-4-hydroxyproline	C01157	5810	HMDB00725	**0.1565**	1.3776
trans-urocanate	C00785	736715	HMDB00301	**0.2652**	1.0539
triacetate lactone	C02752	-	-	**0.5979**	1.2466
trigonelline (N'-methylnicotinate)	C01004	5570	HMDB00875	1.1598	1.0576
trimethylamine N-oxide	C01104	1145	HMDB00925	**0.0264**	1.382
tryptophan	C00078	6305	HMDB00929	**7.4127**	1.3305
tyrosine	C00082	6057	HMDB00158	**2.0077**	0.7596
tyrosol	C06044	10393	HMDB04284	0.3228	2.0773
uracil	C00106	1174	HMDB00300	**0.3727**	0.9969
uridine	C00299	6029	HMDB00296	**8.7017**	1.3099
valine	C00183	6287	HMDB00883	**3.1538**	0.4296
xanthine	C00385	1188	HMDB00292	0.5519	0.8845
xanthopterin	-	8397	-	**0.2739**	1.4042
xanthosine	C01762	64959	HMDB00299	**0.2763**	**1.734**

1. Metabolomics database entries for reported metabolites. KEGG: Kyoto Encyclopedia of Genes and Genomes; PUBCHEM: database maintained by the National Center for Biotechnology Information; HMDB: Human Metabolome Database.
2. Fold difference comparisons of metabolites across selected treatments. Calculation: First, the raw abundance of each metabolite was median-scaled for each individual by dividing their metabolite raw abundance by the median raw abundance for that metabolite across the entire data set . Fold difference was then calculated by dividing the median-scaled abundance of the metabolite in the numerator by the median-scaled abundance of that metabolite in the denominator treatment.
3. Bold indicates a metabolite fold change that differed significantly for the indicated comparison. Italics indicate a metabolite with a statistically-significant interaction between both comparisons.
4. PS: Penicillin-Streptomycin antibiotic treatment; NPS: No Penicillin Streptomycin antibiotic.
5. 20%: Tissues treated with 20% oxygen; 1%: Tissues treated with 1% oxygen.
